# Advantages of petrosectomy for superficial temporal artery to superior cerebellar artery bypass based on three-dimensional distance measurements using cadaver heads

**DOI:** 10.1007/s10143-021-01686-z

**Published:** 2021-11-04

**Authors:** Kenji Uda, Kuniaki Tanahashi, Takashi Mamiya, Fumiaki Kanamori, Kinya Yokoyama, Masahiro Nishihori, Takashi Izumi, Yoshio Araki, Ryuta Saito

**Affiliations:** Department of Neurosurgery, Nagoya University Graduate School of Medicine, 65 Tsurumai-cho Showa-ku Nagoya, Aichi, 466-8550 Japan

**Keywords:** Anterior transpetrosal approach, Bypass, Combined petrosal approach, Subtemporal approach, Superficial temporal artery, Superior cerebellar artery

## Abstract

Superficial temporal artery (STA) to superior cerebellar artery (SCA) bypass is usually performed via the subtemporal approach (StA), anterior transpetrosal approach (ApA), or combined petrosal approach (CpA), but no study has yet reported a quantitative comparison of the operative field size provided by each approach, and the optimal approach is unclear. The objective of this study is to establish evidence for selecting the approach by using cadaver heads to measure the three-dimensional distances that represent the operative field size for STA–SCA bypass. Ten sides of 10 cadaver heads were used to perform the four approaches: StA, ApA with and without zygomatic arch osteotomy (ApA-ZO^−^ and ApA-ZO^+^), and CpA. For each approach, the major-axis length and the minor-axis length at the anastomosis site (La-A and Li-A), the major-axis length and the minor-axis length at the brain surface (La-B and Li-B), the depth from the brain surface to the anastomosis site (Dp), and the operating angles of the major axis and the minor axis (OAa and OAi) were measured. Shallower Dp and wider operating angle were obtained in the order CpA, ApA-ZO^+^, ApA-ZO^−^, and StA. In all parameters, ApA-ZO^−^ extended the operative field more than StA. ApA-ZO^+^ extended La-B and OAa more than ApA-ZO^−^, whereas it did not contribute to Dp and OAi. CpA significantly decreased Dp, and widened OAa and OAi more than ApA-ZO^+^. ApA and CpA greatly expanded the operative field compared with StA. These results provide criteria for selecting the optimal approach for STA-SCA bypass in light of an individual surgeon’s anastomosis skill level.

## Introduction

Superficial temporal artery (STA) to superior cerebellar artery (SCA) bypass is known to be effective for revascularization in steno-occlusive disease of the posterior circulation [2, 3, 5, 8, 10, 11, 16, 22, 27]. This procedure is also required when an aneurysm in the posterior circulation is treated using a flow alteration technique that requires trapping [13, 14, 18, 23]. However, cases that actually require STA-SCA bypass are rare, at 3.6% of all total bypass surgery cases [29]. The subtemporal approach (StA) is most often used for STA–SCA bypass because of its simple procedure and shorter operating time. On the other hand, this approach has the disadvantages that it provides a deep and narrow operative field that makes the bypass difficult to perform and can cause serious intraoperative complications due to cerebral retraction [9, 10]. A few case reports have described the anterior transpetrosal approach (ApA) [8, 12, 23] or the combined petrosal approach (CpA) [7, 16] as helpful in expanding the operative field for STA–SCA bypass. These approaches require anatomical knowledge of the skull base. Despite the rare frequency of STA–SCA bypass, the operator is required to achieve the bypass safely and reliably. Therefore, the approach should be chosen to provide a comfortable operative field that is commensurate with the operator’s skill, while also considering the level of invasiveness. The objective of this study was to establish quantitative evidence for the choice of approach by using cadaver heads to measure the three-dimensional distances that represent the size of the operative field for STA–SCA bypass.

## Materials and methods

**Fig. 1:**
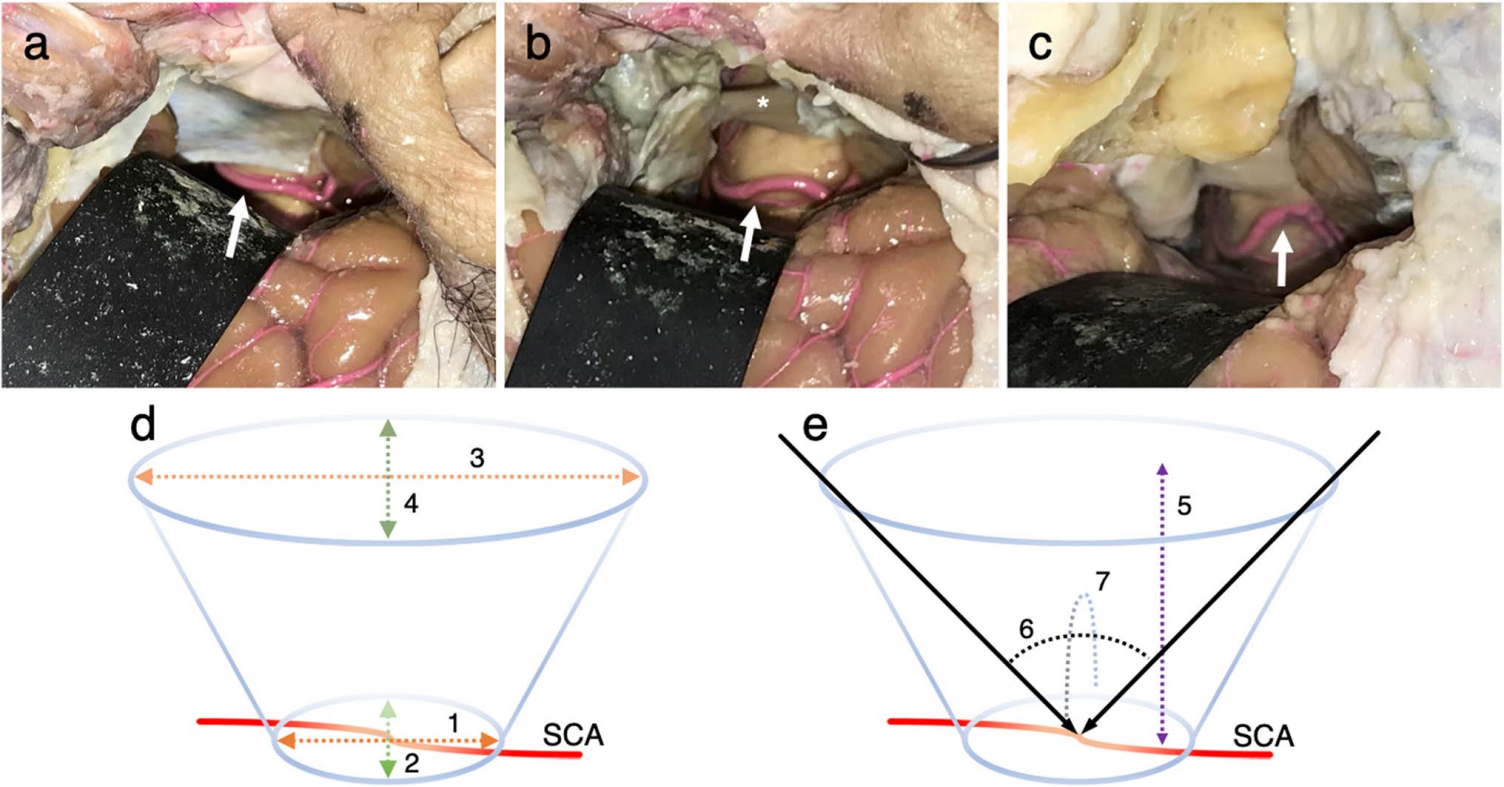
Measurement method for each surgical approach. The white arrows show the superior cerebellar artery (SCA) in the operative field for the subtemporal approach (**a**), the operative field for the anterior transpetrosal approach with zygomatic arch osteotomy (**b**), and the operative field for the combined petrosal approach (**c**). The asterisk shows the trigeminal nerve. **d**, **e** Schematic diagram of measurements. (1) Major-axis length at the anastomosis site. (2) Minor-axis length at the anastomosis site. (3) Major-axis length at the brain surface. (4) Minor-axis length at the brain surface. (5) Depth from the brain surface to the anastomosis site. (6) Operating angle of the major axis. (7) Operating angle of the minor axis

Ten sides of 10 cadaver heads (one side per head) were used. The four approaches of the StA (Fig. 1a), ApA without zygomatic arch osteotomy (ApA-ZO^−^), ApA with zygomatic arch osteotomy (ApA-ZO^+^) (Fig. 1b), and CpA (Fig. 1c), in that order, were performed on one side each following the actual surgical procedure. For each approach, seven parameters were measured: the major-axis length at the anastomosis site (La-A), the minor-axis length at the anastomosis site (Li-A), the major-axis length at the brain surface (La-B), the minor-axis length at the brain surface (Li-B), the depth from the brain surface to the anastomosis site (Dp), the operating angle of the major axis (OAa), and the operating angle of the minor axis (OAi) (Fig. 1d, e). The measurements were made manually with a ruler or protractor. The cadaver heads were fixed in Thiel solution containing formalin and propylene glycol (Thiel’s fixation solution, A.S. Chemical, Osaka, Japan) and injected with colored latex. This research was performed in the clinical anatomy laboratory at the authors’ institution and was approved by the institution’s Ethics Committee (no. 2016–0354-2).

### Surgical approach

The StA was performed first, after which anterior petrosectomy was added to perform the ApA-ZO^−^. Zygomatic arch osteotomy was then conducted to perform the ApA-ZO^+^, and finally mastoidectomy was also conducted to perform the CpA. Each of the surgical procedures is described below.

### Surgical procedure for the StA

**Fig. 2:**
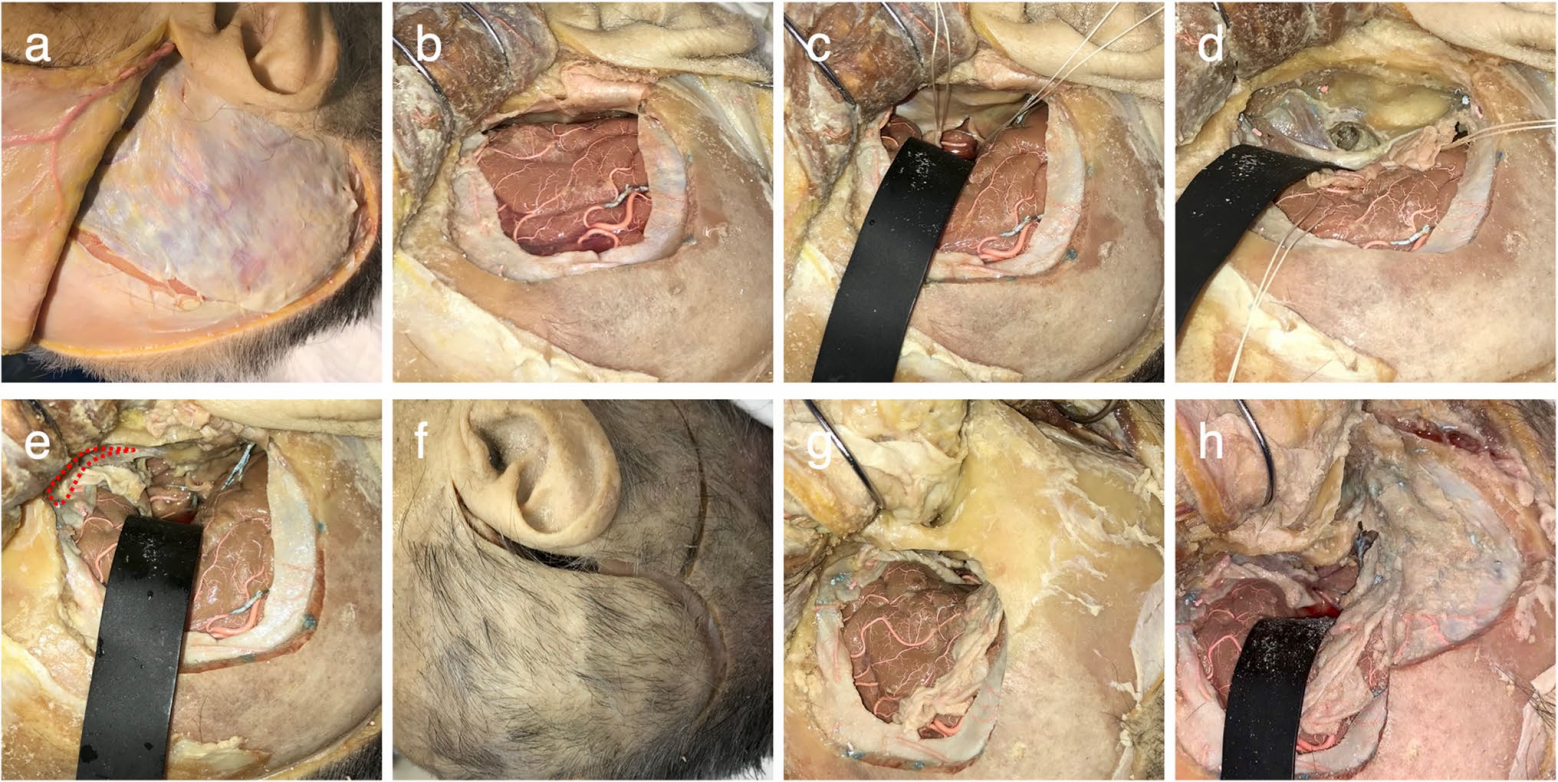
Description of each surgical approach. **a** Skin incision for the subtemporal approach. **b** Craniotomy for the subtemporal approach. **c** The tentorium has been peeled back to the left and right, and the superior cerebellar artery secured. **d** Operative field following anterior petrosectomy. Care is taken to avoid damaging the internal auditory meatus when removing the tip of the petrous bone. **e** The temporalis muscle has been drawn caudad by the zygomatic arch osteotomy, enabling the craniotomy to be extended by 1–2 cm. The additional craniotomy is indicated by the red dotted line. **f** Skin incision for the combined petrosal approach. **g** The skin flap is peeled back as a single layer, and mastoidectomy is performed. **h** Following mastoidectomy, an incision is made in the dura mater, and the superior cerebellar artery is secured. The semicircular canals are preserved

The cadaver head was placed in a 90-degree lateral position. A large question mark–shaped incision was made in the skin (Fig. 2a), beginning from the point below the caudal edge of the zygomatic arch in front of the tragus. The temporalis muscle and fascia were then elevated and reflected anteriorly. A square bone flap, approximately 6 × 6 cm^2^, was then cut, placing one-third behind and two-third in front of the external auditory canal (Fig. 2b). This inferior margin of the craniectomy was flushed with the temporal fossa floor. An arc-shaped incision was then made in the dura mater, after which the temporal lobe was retracted, and the tentorium was cut immediately behind the point at which the trochlear nerve enters in order to reach the SCA. The cut tentorial edges were pulled forward and backward, respectively, using silk thread to prepare the operative field for the bypass (Fig. 2c).

### Surgical procedure for the ApA-ZO^−^

The middle meningeal artery was first divided at the foramen spinosum, after which either the foramen ovale on its anteromedial side or the arcuate eminence (AE) on its posterior side were identified. The dura mater was peeled back until the petrosal ridge was fully exposed, with care taken to avoid damaging the greater superficial petrosal nerve (GSPN). The so-called Kawase rhomboid, comprising the tip of the petrous bone enclosed on four sides by the GSPN, trigeminal impression, AE, and petrous ridge, was then drilled. In this process, care was taken to avoid damaging the internal cerebral artery and cochlea on the medial side of the GSPN and the internal auditory meatus, located on the line bisecting the angle formed between the GSPN and AE (Fig. 2d). The dura maters of the middle and posterior fossa were cut open along the superior petrosal sinus (SPS). After that, an incision was made from the tentorial outer edge to join up with the tentorial incision on the medial side made in the StA and completely divide the tentorium. The dural incision on the posterior side was extended anteriorly along the SPS to open Meckel’s cave, further extending the operative field.

### Surgical procedure for the ApA-ZO^+^

First, the zygomatic process located just below the skin incision was exposed, and the periosteum of the frontal region was inverted to expose the lateral orbital rim. Then, an incision was made in the deep temporal fascia to connect these two parts, exposing the upper edge of the zygomatic arch over its entire length. At this time, care must be taken not to incise the shallow layer and damage the facial nerve. The temporal muscle is freed from its attachment to the inner surface of the zygoma, while the masseter muscles that attach to the medial and inferior edges of the zygomatic arch remain attached. Zygomatic arch osteotomy was performed by carrying out two main cuts. The posterior cut was made just in front of the articular tubercle at a 45-degree angle, and the anterior cut was made at the temporal process vertically behind the marginal tubercle. The zygomatic arch remained attached to temporal muscle and was reflected downwards to prevent masseter muscle atrophy. Retraction downwards of the temporal muscle broadly exposes around 1 cm of the sphenoidal bone, and bone removal in this area was added (Fig. 2e).

### Surgical procedure for the CpA

A postauricular C-shaped scalp incision was made to expose the mastoid (Fig. 2f, g). After a large, L-shaped temporo-occipital and suboccipital craniotomy was made, mastoidectomy was performed, preserving the semicircular canals and fallopian canal. Incisions were made in the dura maters of the middle and posterior fossa along the SPS at least 5 mm anterior to the sigmoid sinus and transverse sinus junction, and the tentorium was identified. A new incision was made in the tentorium toward the midline. The presigmoid dura was then opened toward the jugular bulb in an L-shaped fashion, broadly exposing the SCA (Fig. 2h).

### Statistical analysis

To compare the sizes of operative fields in the same cadaver head, continuous variables for two groups were analyzed using a paired *t*-test. Statistical data were analyzed using EZR [15], with significance set at the *P* < 0.05 level.

## Results

Tables 1 and 2 show the measurements (expressed as means ± standard deviations) made during the four approaches, StA, ApA-ZO^−^, ApA-ZO^+^, and CpA. La-A measured 17.4 mm, 19.6 mm, 20.5 mm, and 20.8 mm, respectively, and Li-A measured 12.6 mm, 15.6 mm, 15.8 mm, and 15.5 mm, respectively, smallest for the StA and equivalent for the other three approaches. La-B was 32.9 mm, 35.7 mm, 38.7 mm, and 35.9 mm, respectively, smallest for the StA and largest for the ApA-ZO^+^. Li-B was 14.8 mm, 19 mm, 18.5 mm, and 18.1 mm, respectively, smallest for the StA and equivalent for the other three approaches. Dp was 40 mm, 37.2 mm, 37 mm, and 34.6 mm, respectively, deepest for the StA and shallowest for the CpA. OAa was 38.2°, 45.2°, 54.3°, and 58.2°, respectively, and OAi was 21.3°, 29.0°, 30.4°, and 39.3°, respectively, narrowest for the StA and widest for the CpA.

**Table 1:** Length measurements of the anastomosis site and brain surface made in the four approaches

Approach	StA	ApA-ZO^−^	ApA-ZO^+^	CpA
	Mean ± SD	Mean ± SD	Mean ± SD	Mean ± SD
Major-axis length at anastomosis site (La-A) (mm)	17.4 ± 3.4	19.6 ± 3.5	20.5 ± 3.9	20.8 ± 3.3
Minor-axis length at anastomosis site (Li-A) (mm)	12.6 ± 1.4	15.6 ± 1.9	15.8 ± 2.5	15.5 ± 1.6
Major-axis length at brain surface (La-B) (mm)	32.9 ± 4.4	35.7 ± 3.7	38.7 ± 3.5	35.9 ± 3.4
Minor-axis length at brain surface (Li-B) (mm)	14.8 ± 2.8	19.0 ± 2.7	18.5 ± 2.1	18.1 ± 2.4

*Abbreviations*: *StA* subtemporal approach, *ApA-ZO*^*−*^ anterior transpetrosal approach without zygomatic arch osteotomy, *ApA-ZO*^+^ anterior transpetrosal approach with zygomatic arch osteotomy, *CpA* combined petrosal approach, *La-A* major-axis length at the anastomosis site, *Li-A* minor-axis length at the anastomosis site, *La-B* major-axis length at the brain surface, *Li-B* minor-axis length at the brain surface

**Table 2:** Measurements of depth and operating angle made in the four approaches

Approach	StA	ApA-ZO^−^	ApA-ZO^+^	CpA	StA vs ApA-ZO^−^	ApA-ZO^−^ vs ApA-ZO^+^	ApA-ZO^+^ vs CpA
	Mean ± SD	Mean ± SD	Mean ± SD	Mean ± SD	Paired *t*-test	Paired *t*-test	Paired *t*-test
Depth from the brain surface to the anastomosis site (Dp) (mm)	40.0 ± 5.3	37.2 ± 6.5	37 ± 6.7	34.6 ± 5.9	*p* = .0061**	n.s	*p* = .011*
Operating angle of the major axis (OAa) (degrees)	38.2 ± 4.5	45.2 ± 4.8	54.3 ± 5.8	58.2 ± 4.1	*p* = .0052**	*p* = .00048**	*p* = .046*
Operating angle of the minor axis (OAi) (degrees)	21.3 ± 4.2	29.0 ± 4.6	30.4 ± 7.9	39.3 ± 11.5	*p* = .0044**	n.s	*p* = .0032**

*Abbreviations*: *StA* subtemporal approach, *ApA-ZO*^*−*^ anterior transpetrosal approach without zygomatic arch osteotomy, *ApA-ZO*^+^ anterior transpetrosal approach with zygomatic arch osteotomy, *CpA* combined petrosal approach, *Dp* depth from the brain surface to the anastomosis site, *OAa* operating angle of the major axis, *OAi* operating angle of the minor axis, *SD* standard deviation, *n.s.* not significant

^*^p < 0.05; **p < 0.01

Differences between each surgical approach were then compared statistically. A comparison between the StA and the ApA-ZO^−^ showed that the ApA-ZO^−^ widened OAa and OAi, and decreased Dp significantly more than StA. A comparison between the ApA-ZO^−^ and the ApA-ZO^+^ showed that the ApA-ZO^+^ widened OAa significantly more than the ApA-ZO^−^, whereas there were no significant differences in Dp and OAi. A comparison between the ApA-ZO^+^ and the CpA showed that CpA decreased Dp and widened OAa and OAi significantly more than the ApA-ZO^+^.

## Discussion

The size of the operative field for the STA–SCA bypass via four different approaches was measured using cadaver heads, and it was found that shallower Dp and a wider operating angle were obtained in the order CpA, ApA-ZO^+^, ApA-ZO^−^, and StA. A comparison between the StA and the ApA-ZO^−^ showed that the ApA-ZO^−^ greatly expanded the operative field compared with that provided by the StA, and a comparison between the ApA-ZO^−^ and the ApA-ZO^+^ showed that zygomatic arch osteotomy contributed to a widened OAa, whereas it did not contribute to Dp, OAi, and the minor-axis length, such as Li-A and Li-B. A comparison between the ApA-ZO^+^ and the CpA showed that Dp was shallower and the operating angles were wider for the CpA, although there was no great difference in the operative field size itself. The CpA is slightly invasive and time-consuming, whereas the ApA can be performed easily in a familiar surgical field via fronto-temporal craniotomy. For these reasons, it is possible that the ApA is generally the most reasonable approach for STA–SCA bypass that balances invasiveness with securing a sufficiently wide operative field. It is well known that petrosectomy expands the operative field compared with StA alone, but no reports performed a quantitative comparison of the operative field provided by each approach, and, therefore, it has not been possible to discuss how effective petrosectomy is. Although cases requiring STA-SCA bypass are rare, the surgeon is required to achieve the bypass reliably. Therefore, the selection of approach that provides an appropriate operative field size for each surgeon is important. The results of this study provide one of the criteria for selecting the optimal approach to achieve an STA-SCA bypass safely and reliably, in light of an individual surgeon’s anastomosis skill level.

### Comparison between the StA and the ApA-ZO^−^

**Fig. 3:**
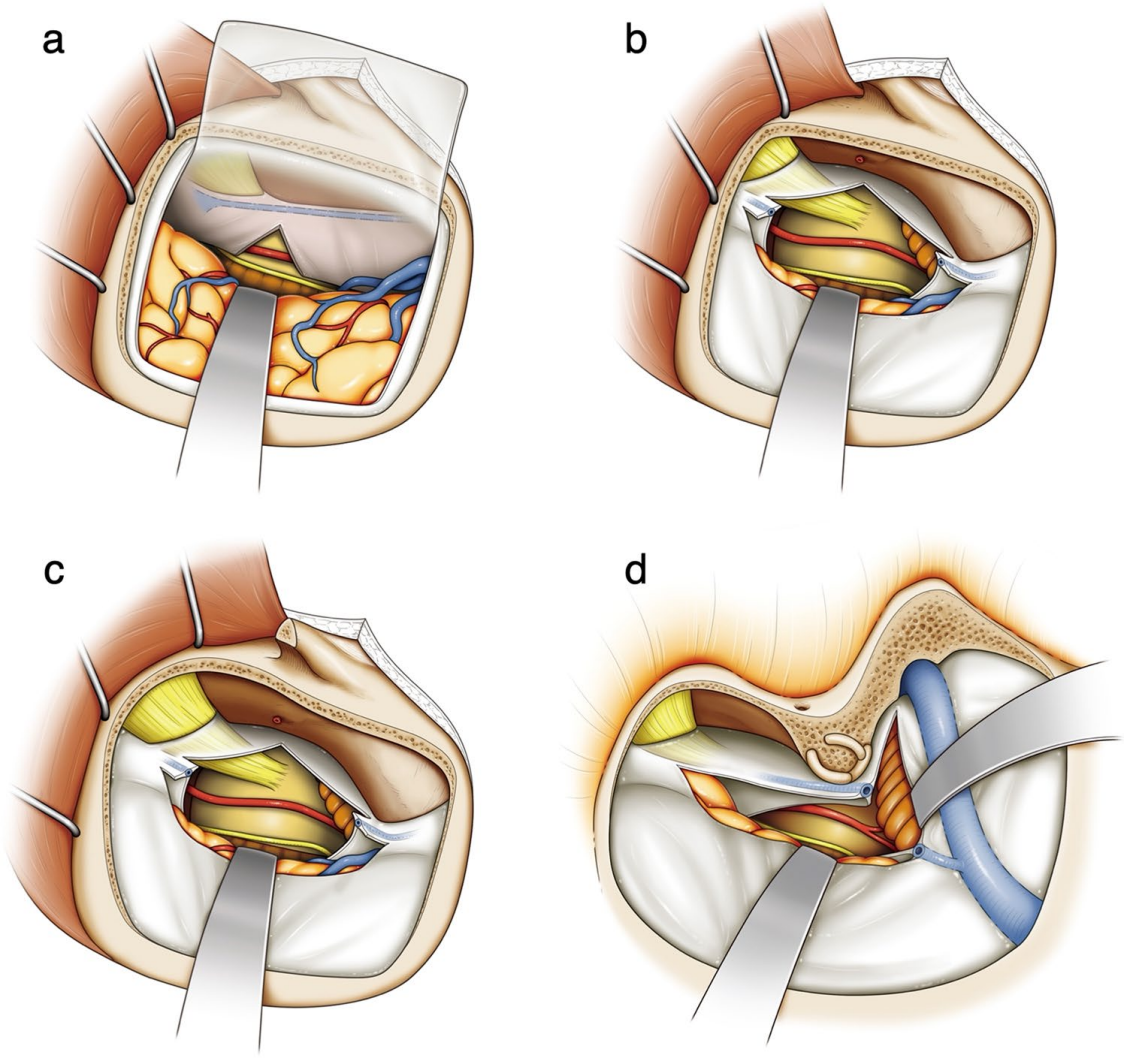
Illustration of each surgical approach. **a** Illustration of the subtemporal approach. Because the surgical field is narrow and deep, retraction of the temporal lobe becomes tense, resulting in a high risk of brain damage. A tentorial incision is made posteriorly to the entry point of trochlear nerve. **b** Illustration of the anterior transpetrosal approach without zygomatic arch osteotomy. By completely cutting off the tentorium, the surgical field expands in the major-axis direction. In addition, the surgical field expands in the minor-axis direction by peeling the dura mater off the skull base and excising the tip of the petrosal bone. The cutting point of the tentorium is the same as the subtemporal approach. Since the temporal lobe is retracted over the dura, the risk of brain damage is less. **c** Illustration of the anterior transpetrosal approach with zygomatic arch osteotomy. The field of the craniotomy is enlarged, and the major-axis length at brain surface is extended, but the minor-axis length at the brain surface does not change with the zygomatic arch osteotomy. In addition, the depth and the size of the anastomosis site do not increase. **d** Illustration of the combined petrosal approach. Mastoidectomy expands the surgical field in the minor-axis direction and makes the depth shallower. The tentorium is cut off posteriorly to the anterior transpetrosal approach

In general, the StA is often used to perform an STA–SCA bypass [2, 17], whereas the operative field is horizontally long and narrow, as well as deep, making it extremely confined (Fig. 3a). This leads to excessive brain retraction during anastomosis, resulting in a high incidence of serious complications such as cerebrovascular damage and temporal lobe contusion. Previous reports have shown that the STA to SCA and posterior cerebral artery bypass in the StA has a high rate of complications (rates of serious complications and mortality were 20% and 12%, respectively) and a relatively low patency rate of 79% [9]. Although the procedure has improved in recent years, it is hard to say that the StA can be safely performed by any operator. The ApA is achieved by performing the StA with the additional removal of the tip of the petrous bone and division of the tentorium. In this study, it was shown that the ApA expands the operative field greatly compared with that of the StA. In both approaches, the extent of the craniotomy, the position where the tentorium is cut, and the target anastomosis site of the SCA are the same. However, removing the tip of the petrous bone and dividing the tentorium completely, as well as detaching the dura mater from the bone, increased the mobility of the brain when it was retracted, both enlarging the operative field and reducing the distance to the anastomosis site (Fig. 3b). In addition, the temporal lobe and the vein of Labbe are protected by the dura mater during the surgical procedure in the ApA, which tends to reduce brain contusion when compared to the temporal lobe that would be retracted intradurally in the StA. On the other hand, it should be noted that the ApA also has disadvantages that include the possibility of facial nerve injury and hearing impairment, CSF leakage, and slight prolongation of operating time.

### Comparison between the ApA-ZO^−^ and the ApA-ZO^+^

The effects of removing the zygomatic arch are still under debate [1, 6]. The present study showed that zygomatic arch osteotomy draws the temporalis muscle further caudally, making it possible to extend the bone removal by 1–2 cm, and that La-B and OAa can be expanded (Fig. 3c). In the STA-SCA bypass, the expansion of the operative field in the major axis, such as La-B and OAa, is effective for the stay suture procedure. On the other hand, most of the anastomotic procedures other than the stay suture are movements in the minor-axis direction; therefore, the size of the operative field in the minor axis, such as OAi, Li-A, and Li-B, is even more important. For that reason, it seems that the effectiveness of the zygomatic arch osteotomy for the STA-SCA bypass, which does not contribute to minor-axis length, is limited. Moreover, since zygomatic arch osteotomy has been reported to cause complications including facial nerve palsy, trismus, sensory disturbance in the trigeminal nerve territory, and masseter muscle atrophy, its use must be carefully considered [21, 24].

### Comparison between the ApA-ZO^+^ and the CpA

In the present study, the CpA provided the shallowest Dp and widest OA among the four approaches; therefore, the STA-SCA bypass itself is the easiest to perform in the CpA (Fig. 3d). However, the CpA requires skull base surgical techniques such as mastoidectomy that are unfamiliar to general neurosurgeons and exposure of the sigmoid sinus and other structures to which damage could be fatal. Mastoidectomy has the risk of damage to the semicircular canals causing hearing loss, facial nerve damage, and postoperative cerebrospinal leakage [7, 25]. The CpA is also relatively time-consuming and highly invasive. Given the above, it seems difficult to select the CpA just for the STA-SCA bypass in general. On the other hand, since the length of the operative field is comparable to that of the CpA in the ApA, the ApA could be the most reasonable approach for STA–SCA bypass considering the balance between the complexity of the procedure and the size of the operative field.

### Use of the ApA + ZO^+^ for internal maxillary artery to SCA bypass

In recent years, high-flow bypass to the posterior circulation using the internal maxillary artery (Imax) as the bypass donor with a radial artery graft (RAG), saphenous vein graft, or STA graft has been attracting attention [4, 19, 20, 26, 28, 30]. In this technique, the high-flow bypass to the posterior circulation can be performed via the StA or ApA, both of which are familiar approaches. This technique does not need neck exposure, and a graft distance of about 50 mm may be sufficient [30], which can be a less-invasive substitute. The Imax can easily be secured beneath the temporalis muscle when zygomatic arch osteotomy is performed, and the ApA + ZO^+^ may thus be a good match for this procedure.

### Limitations

Anatomical cadaveric studies have several limitations. First, the optimal approach should be evaluated based not just on the size of operative field, but also on the patency rate of the bypass and the morbidity of the approach. However, these factors are outside the scope of a cadaveric study. There have been few reports about STA-SCA bypass with petrosectomy; therefore, it is necessary to gather more cases for actual results. Second, it was not possible to recreate some of the anatomical features that could change in clinical cases, including brain relaxation or neurovascular distortion due to aneurysms. Thus, approaches may be limited by the site or shape of the lesion.

## Conclusions

The ApA and CpA greatly expanded the operative field compared with the StA. The results of this study provide criteria for selecting the optimal approach to achieve an STA-SCA bypass safely and reliably, in light of an individual surgeon’s anastomosis skill level.

## Data Availability

The data that support the findings of this study are available from the corresponding author upon reasonable request.
